# Survival and Disease Progression in Older Adult Patients With Cirrhosis: A Retrospective Study

**DOI:** 10.1155/2024/5852680

**Published:** 2024-08-08

**Authors:** Khaled Al-Smadi, Ammar Qureshi, Michelle Buitrago, Besher Ashouri, Zeid Kayali

**Affiliations:** ^1^ Department of Gastroenterology and Hepatology University of California-Riverside School of Medicine, Riverside, USA; ^2^ Department of Biology State University of New York – Stony Brook, Stony Brook, USA

**Keywords:** cirrhosis, MELD Na, older adult patients, survival

## Abstract

**Background:** Cirrhosis incidence in older adult patients has been increasing with limited data on their survival. This study is aimed at investigating the survival and disease progression in older adult patients with cirrhosis compared to younger patients.

**Methods:** This is a retrospective single-center study. Patients aged above 50 with a confirmed diagnosis of cirrhosis based on biopsy, FibroSure test, splenomegaly, and low platelets < 120 × 10^9^/L) or imaging findings including FibroScan were included. Patients with active substance abuse, transjugular intrahepatic portosystemic shunt (TIPS), prior spontaneous bacterial peritonitis (SBP), variceal hemorrhage, model for end-stage liver disease-Na (MELD − Na) ≥ 20, had liver transplantation, malignancy except for squamous cell carcinoma, and other comorbidities such as congestive heart failure (CHF), chronic obstructive pulmonary disease (COPD), and end-stage kidney disease with glomerular filtration rate (GFR) < 30 were excluded. Patients' records from the liver clinic were reviewed and demographics, laboratory, and compensation and decompensation status were collated. Patients were separated into two groups based on age 50–64 years and age ≥ 65. The primary endpoint was death, and the secondary endpoint was disease progression measured by the baseline to 12-month increase in MELD-Na score. The Kaplan–Meier analysis was conducted to compare the survival between the two groups. Cox regression analysis was performed to identify independent risk factors for poor survival.

**Results:** A total of 191 patients diagnosed with cirrhosis met the inclusion and exclusion criteria. There were 80 patients aged 50–64 years and 111 patients aged ≥ 65 years. Significantly shorter survival times were seen among patients aged ≥ 65 years compared to those aged 50–64 years (73.3 ± 4.8 vs. 151.5 ± 22.7; *p* < .001). Age of diagnosis ≥ 65 years (*p* < 0.001), male gender (*p* = .013), body mass index (BMI) < 30 (*p* = 0.005), and decompensation (*p* = 0.008) were found to be independent risk factors for poor survival. MELD-Na scores increased significantly in 12 months of follow-up from baseline, but only in patients with decompensated cirrhosis (*p* = 0.013).

**Conclusions:** Cirrhotic patients aged ≥ 65 years have significantly poor survival compared to younger patients. A prospective study is needed to further investigate the effect of age and obesity on survival and disease progression in older adult patients with cirrhosis.

## 1. Introduction

Cirrhosis is a terminal disease and liver transplantation is the only curative treatment [1]. Decompensation and hepatocellular carcinoma (HCC) represent the most ominous outcome. Disease progression is usually manifested by the occurrence of one of the decompensation features such as ascites, variceal hemorrhage, or hepatic encephalopathy, and it can be measured by calculating the model for end-stage liver disease-Na (MELD-Na) score. Decompensated cirrhosis patients have significantly shorter survival time compared to patients with compensated cirrhosis [2].

Multiple factors affect the rate of disease progression and mortality. This includes duration of cirrhosis, underlying chronic liver disease, ongoing substance abuse, age, and gender [3, 4]. The effect of aging on cirrhosis progression became a matter of interest due to the fact that the prevalence of cirrhosis in older adult patients has been increasing, and it is expected to continue to increase for the next decades due to the increasing rates of obesity and metabolic associated-steatotic liver disease (MASLD) and the number of aging populations [5–7].

As the incidence of cirrhosis is increasing in the older adults, it is expected that cirrhosis will have a significant burden on the healthcare system due to increased hospitalizations and care from decompensated disease in older adult patients [8, 9].

The effect of aging on survival in older adults with cirrhosis has been attributed mainly to the fact that the majority of these patients are not transplant candidates due to either their age and/or comorbidity. Additionally, there are age-related physiological changes in the liver including decreased hepatic volume, weakened intrahepatic immunity, and reduced regeneration potential [10], which will accelerate disease progression and mortality in this population.

Furthermore, older adult patients tend not to tolerate diuretics due to other comorbidities such as diabetes-related kidney disease and low muscle mass which can lead to more frequent hospitalizations for the management of ascites [11, 12]. Very few studies have shown that age is one of the factors affecting the survival in older patients. These studies had major limitations such as a lack of clear stratification of the subjects according to their ages, not being powered enough to study the effect of aging on survival or it included patients with more advanced disease or comorbidity [5, 13–15].

There is a need for more studies with clear age stratification to determine the effect of aging on cirrhosis outcomes in the older adult patients and address thoroughly all the potential factors that might affect the survival and mortality in well-stratified and less advanced groups of patients. Accordingly, this study is aimed at comparing the survival and disease progression of compensated and decompensated cirrhosis in older adult patients compared to younger patients.

## 2. Methodology

### 2.1. Study Design

This is a retrospective study, using the existing data from a single-center liver clinic, which is aimed at investigating the outcome of aging on disease progression and survival in older adult patients diagnosed with cirrhosis. The primary end point is 84-month survival, and the secondary end point is disease progression as measured by MELD-Na at 12 months of follow-up.

### 2.2. Patients

The University of California Riverside liver clinic records were reviewed retrospectively between the years 2015 and 2023. Patients with compensated and decompensated cirrhosis were included regardless of cirrhosis etiology. Only patients aged 50 years and above were included. The diagnosis of cirrhosis was made based on documented findings of liver biopsy, splenomegaly, and thrombocytopenia (platelets < 120 × 109/L), FibroScan (elasticity > 20 kPa), Fibrotest, or evidence of ascites on imaging or physical examination. Patients with other comorbidities that might impact survival such as chronic obstructive lung disease (COPD), congestive heart failure (CHF), malignancy other than squamous cell carcinoma and glomerular filtration rate < 30 were excluded. Patients with MELD − Na ≥ 20, TIPS, SBP, history of esophageal or gastric variceal bleeding, or who were actively using substances were excluded as well.

Decompensation was diagnosed based on the presence of ascites and hepatic encephalopathy. The diagnosis of ascites was based on imaging, prior paracentesis, or physical exam documenting abdominal distension with a fluid wave. Encephalopathy was determined according to the West Haven encephalopathy scale with any patients with Stage 1 and above [16]. Patients with a history of variceal bleeding, although considered decompensated, were excluded because we were unable to obtain outside hospital records to confirm the bleeding incidence. A patient who has at least one incidence of decompensation was considered decompensated even if the patient became compensated later while using diuretics or lactulose. The study was approved by the Institutional Review Board.

### 2.3. Patients' Follow-Up

The follow-up from baseline was defined by the estimated time of cirrhosis diagnosis in compensated patients and by the estimated time of first decompensation in the decompensated patients. The duration of follow-up was 84 months. There was no crossover follow-up between the two groups defined by age ≥ 65 years and 50–64 years. Disease progression was estimated based on the difference between MELD-Na scores calculated at baseline and 12 months. The primary end point was death, and the secondary end point was disease progression within 12 months. Patients' demographics, laboratory, and medical history were collected from electronic health records and during follow-up accordingly. The estimated time of death was determined from the clinic records or via a phone call to the patient's family for patients who were lost to follow-up or for whom the survival could not be determined from the clinic records.

### 2.4. Statistical Analysis

Statistical analysis was performed using IBM SPSS Statistics version 26 (IBM Corp., Armonk, NY). Patients' demographics and medical history variables were compared by age groups using the Pearson chi-square tests. Variables with multiple categories were dichotomized (each vs. all others) for subgroups. The Mann–Whitney *U* tests were used to compare baseline laboratory results and the estimated duration of cirrhosis by age groups. Two survival distributions were generated using the Kaplan–Meier method for subgroups based on age. Log-rank tests were run to determine the significance of age differences in the survival distributions. A hierarchical stepwise Cox regression analysis was conducted to develop a set of independently significant covariates that could be used to predict survival in the study cohort. Covariates were entered into the model in a stepwise fashion using an alpha of.05 as the entry criterion. In the first block, covariates were selected from demographic characteristics including Hispanic ethnicity, White ethnicity, age group, and male gender. The pool of covariates in the second block included patients' disease and medical characteristics as well as baseline laboratory results as listed in Tables 1 and 2. Again, covariates were selected for model inclusion in a stepwise fashion and were entered into the model only if they added significantly to the prediction of survival above and beyond covariates already entered. Log minus log functions for each significant covariate in the final model were plotted against time to confirm that the underlying assumption of proportionality had been met. A hierarchical stepwise regression was conducted to predict disease progression, expressed as the increase in MELD-Na scores from baseline to 12-month follow-up. Potential predictors included all patient characteristics listed in Table 1. As a follow-up to the regression result, paired *t*-tests were computed to illustrate the differences in disease progression within compensated and decompensated patients.

## 3. Results

A total of 191 patients met the inclusion and exclusion criteria of the study. There were 111 patients aged ≥ 65 years group (median age 69 years) and 80 patients aged 50–64 years (median age 56 years). Patients' demographics and laboratory findings are presented in Tables 1 and 2.

Older adult patients aged ≥ 65 years had a higher rate of diabetes and MASLD cirrhosis compared to younger patients. Younger patients had a higher rate of alcoholic related disease compared to the older adult patients. The incidences of chronic kidney disease and cardiac disease were very low in the younger patients (2.5%), compared to the older adult patients (11-14%). (Table 1.) Baseline ALT (alanine aminotransferase) was higher in the younger patients (Table 2).

The estimated duration of cirrhosis did not differ significantly by age. The duration in patients under 65 years of age was 51.8 ± 37.1 months and 44.4 ± 27.5 months for those 65 years of age and older (*p* = 0.306; Table 3).

### 3.1. Survival Analysis

**Figure 1:**
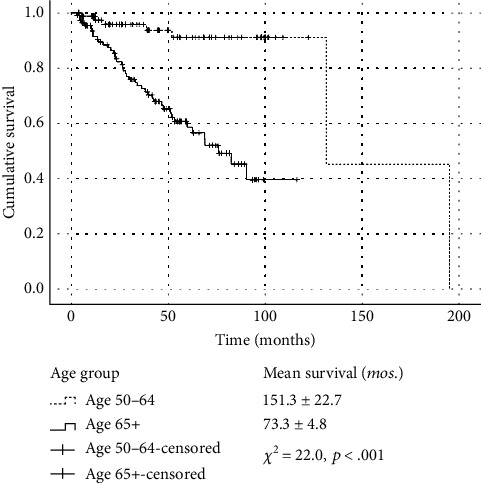
Kaplan–Meier survival distributions stratified by age group.

The Kaplan–Meier analysis showed that older patients (≥ 65 years) experienced significantly shorter survival times compared to patients aged 50–64 years (73.3 ± 4.8 months and 151.3 ± 22.7 months, respectively; *p* < 0.001). (Figure 1). In 48 months, 18% of patients aged ≥ 65 years died compared to only 6% of the group aged 50–64 years.

Stepwise Cox regression analysis showed age ≥ 65 years at diagnosis (HR = 6.9, 95% CI = 2.7 − 17.4, *p* < 0.001), male gender (HR = 2.1, 95% CI = 1.2 − 3.7, *p* = 0.013), BMI < 30 (HR = 2.6, 95% CI = 1.3–5.1, *p* < 0.005) and status of decompensation (HR = 2.2, 95% CI = 1.2–3.8, *p* = 0.008) were all independent risk factors for poor survival (Table 4).

A hierarchical stepwise regression on disease progression from baseline to 12-month follow-up using patient characteristics in Table 1 as potential predictors revealed decompensation as the only significant predictor (*β* = 0.25, *p* = 0.005). To further illustrate this result, paired *t*-tests were conducted within the compensated and decompensated patients (Table 5). Disease progression in the compensated cirrhosis patient group was not significant (baseline and 12-month MELD Na scores were 10.9 and 10.6, respectively; *p* = 0.511). In the decompensated group, there was a significant increase in model-Na scores from baseline to 12-month follow-up (from 13.2 to 15.5; *p* = 0.013).

## 4. Discussion

Aging can lead to physiologic and pathophysiologic changes which might accelerate the progression of chronic liver diseases or decrease tolerability to treatment. These changes include decreased volume and blood flow, a decrease in cytochrome P450 activity, and lowered immune response to pathogens [10]. Aging may be associated with extrahepatic changes such as metabolic syndrome complications, other end-organ diseases, and muscle wasting which can play an important role in decreasing survival and increasing morbidity from existing liver disease in older adult patients [12]. Establishing a clear understanding of the impact of these factors on the progression of cirrhosis in older adult patients is paramount to improving outcomes in this patient population.

Very few studies have looked at the effect of aging as a prognostic factor for survival in patients with cirrhosis, and these studies have included a wide range of adult patients older than 18 and did not stratify patients according to their age groups, and included much advanced cirrhosis [17, 18]. Our study is unique in that it measured survival in patients aged 50 years and above. Identifying the age at which a human is considered an older adult is a matter of dispute. We selected the age of 65 as a definition of the older adult as this age is considered a relative or absolute contraindication for liver transplantation in many centers across the world. Overall, our study results are aligned with the study reported by Abu-Freha et al. which showed that older adult patients aged > 65 have poor survival compared to younger patients [15]. However, in contrast to that study, our study excluded patients with other serious portal hypertension complications such as esophageal varices bleeding, SBP, and patients on the liver transplantation list. This design allowed us to minimize the selection bias by including high-risk patients with more serious complications from cirrhosis.

Our study shows that age ≥ 65, male gender, BMI < 30, and decompensation are independently significant factors affecting survival in patients with cirrhosis. Similar to what has been shown in other studies, cirrhotic males have a higher rate of mortality [19]. In our cohort, this can be explained because of the higher rate of prior alcohol drinking, poor social habits like smoking, and poor access to health care frequently seen in males compared to females.

Our study is the first to show BMI < 30 in cirrhotic patients to be associated with poor survival. This can be explained by the fact that obesity is more frequent in younger populations, and older adult patients tend to have lower BMI due to sarcopenia (muscle wasting). The number of patients in this study was not adequate to stratify the cohort according to their BMI level. We do not believe that morbid obesity plays a role in improving survival, and more studies are needed to look at the effect of sarcopenia with and without obesity on survival in older adult patients. Furthermore, our study was not powered statistically to show the impact of all metabolic syndrome components on survival.

Older adult patients in this study had relatively high 2-year mortality (18%) compared to younger patients (4%). This is aligned with historical studies [19–21].

Our study confirms the paradigm shift in the etiology of cirrhosis in the older adults as most of our patients had MASLD cirrhosis and very few had HCV cirrhosis. The etiology of cirrhosis, however, was not an independent risk factor for survival time in our study. This is contrary to what other studies have shown, that alcoholic cirrhosis and MASLD are independent factors for survival [22, 23].

The ethnicity of patients was not a predictive factor for poor survival or disease progression. This is despite previous reports revealing variations in chronic liver disease-related death among different ethnic groups. The majority of the patients in our study were Hispanic, and ethnicity was evenly distributed within the age groups. The disparities in healthcare outcomes in certain ethnic groups such as Hispanics might play a role in impacting the survival of cirrhotic patients. Our study, however, was not powered to investigate this factor, and it warrants further investigation through larger prospective studies [24].

One of the major limitations of our study, in addition to being retrospective, is that we could not determine the cause of death as these records were not available in the liver clinic records. For this reason, we could not determine all unmeasured variable factors that might have contributed to the mortality in both patient groups such as falls, poor diuretics tolerability, renal failure, and sepsis. Another study limitation is that we did not include variceal bleed as a component for decompensation because we did not have records documenting and verifying the incidence. Variceal bleeding can occur in both compensated and decompensated cirrhosis, regardless of patient age; hence, eliminating this group of patients did not introduce a selection bias from this prospective.

## 5. Conclusion

Our study is one of the very few that has shown poor survival in older adult patients with cirrhosis. The impact of aging on the progression of cirrhosis is a very complex phenomenon that requires more investigation. As the aging population is growing rapidly, more prospective studies are needed to find the impact of intra and extrahepatic factors on disease progression in compensated and decompensated cirrhotic patients and establish better guidelines for how we treat and care for this population.

## Figures and Tables

**Table 1:** Patient characteristics.

**Characteristic**	**Age 50–64**	**Age 65+**
	**n** **(%)**	**n** **(%)**
Ethnicity		
Asian	1 (1.3)	3 (2.7)
Black	6 (7.5)	11 (9.9)
Hispanic	58 (72.5)	68 (61.3)
Middle-eastern	1 (1.3)	3 (2.7)
White	14 (17.5)	26 (23.4)
Male Gender	40 (50.0)	49 (44.1)
Decompensation	29 (36.3)	43 (38.7)
Diabetes mellitus	29 (36.3)	58 (52.3)^∗^
Chronic kidney disease	2 (2.5)	13 (11.7)^∗^
BMI ≥ 30	35 (43.8)	42 (37.8)
Cardiac disease	2 (2.5)	16 (14.4)^∗∗^
Hepatocellular carcinoma	7 (8.8)	10 (9.0)
Cirrhosis cause		
Alcoholic liver disease	31 (38.8)	24 (21.6)^∗∗^
Autoimmune hepatitis	6 (7.5)	4 (3.6)
Cryptogenic	3 (3.8)	7 (6.3)
Hemochromatosis	1 (1.3)	0 (0.0)
Hepatitis C	12 (15.0)	22 (19.8)
MASLD	26 (32.5)	54 (48.6)^∗^
PBC	1 (1.3)	0 (0.0)
Diagnostic modality		
Biopsy	7 (8.8)	8 (7.2)
Cirrhosis on imaging	64 (80.0)	83 (74.8)
FibroScan	7 (8.8)	15 (13.5)
Fibrotest	2 (2.5)	5 (4.5)

*Note:χ* ^2^ by age. FibroScan diagnosis of cirrhosis (elasticity > 20 kPa). Fibrotest diagnosis of cirrhosis (score > 0.74 − 1).

Abbreviations: BMI, body mass index; MASLD, metabolic associated-steatotic liver disease; PBC, primary biliary cholangitis.

^∗^ *p* < .05.

^∗∗^ *p* < .01.

**Table 2:** Baseline laboratory results.

	**Age 50–64 (** **n** = 80**)**	**Age 65+ (** **n** = 111**)**
	**M** **e** **a** **n** **±** **SD**	**M** **e** **a** **n** **±** **SD**
MELD NA	12.3 ± 5.1	11.8 ± 4.1
Albumin	3.6 ± 0.7	3.6 ± 0.5
AST	70.5 ± 154.7	44.5 ± 34.9
ALT	44.1 ± 57.3	32.4 ± 39.2^∗^
ALP	160.2 ± 172.4	134.6 ± 66.4

Abbreviations: ALP, alkaline phosphatase; ALT, alanine aminotransferase; AST, aspartate aminotransferase; MELD-NA, model for end-stage liver disease-sodium.

^∗^ Mann–Whitney *U* test *p* < .05.

**Table 3:** Estimated duration of cirrhosis (months).

**Age**	**N**	**M** **e** **a** **n** ± **SD**	**Range**	**Median**	**p** ^∗^
50–64	80	51.8 ± 37.1	4.0–194.6	45.0	0.306
65+	111	44.4 ± 27.4	2.8–116.6	43.6	
Total	191	47.5 ± 32.0	2.8–194.6	44.8	

^*^Mann–Whitney *U* test by age.

**Table 4:** Hierarchical stepwise Cox regression.

**Step**	**Entered**	**χ** ^2^ **change**	**p** **change**	**HR**	**95% CI**	**p**
1	Age at diagnosis (50–64 vs. ≥65)	26.04	< 0.001	6.9	2.7–17.4	< 0.001
2	Male gender	6.12	0.013	2.1	1.2–3.7	0.013
3	BMI < 30	8.60	0.003	2.6	1.3–5.1	0.005
4	Decompensation	6.75	0.009	2.2	1.2–3.8	0.008

Abbreviations: BMI, body mass index.

**Table 5:** Paired *t*-tests on disease progression from baseline to 12 months within compensated and decompensated patients.

**MELD Na**	**M** **e** **a** **n** ± **SD**	**t** **(df)**	**p**
Within compensated patients (*n* = 77)			
Baseline	10.91 ± 4.2	0.66 (76)	0.511
12 months	10.61 ± 4.3		
Paired differences	0.30 ± 4.0		
Within decompensated patients (*n* = 45)			
Baseline	13.22 ± 4.0	2.59 (44)	0.013
12 months	15.47 ± 5.7		
Paired differences	2.24 ± 5.8		

Abbreviations: MELD-NA, model for end-stage liver disease-sodium.

## Data Availability

Data to support this study's findings are available upon request from the corresponding author.
